# An Animal Model of Acute and Chronic Chagas Disease With the Reticulotropic Y Strain of *Trypanosoma cruzi* That Depicts the Multifunctionality and Dysfunctionality of T Cells

**DOI:** 10.3389/fimmu.2019.00918

**Published:** 2019-04-26

**Authors:** Jose Mateus, Paula Guerrero, Paola Lasso, Claudia Cuervo, John Mario González, Concepción J. Puerta, Adriana Cuéllar

**Affiliations:** ^1^Grupo Inmunobiología y Biología Celular, Facultad de Ciencias, Pontificia Universidad Javeriana, Bogotá, Colombia; ^2^Grupo de Enfermedades Infecciosas, Facultad de Ciencias, Pontificia Universidad Javeriana, Bogotá, Colombia; ^3^Grupo de Ciencias Básicas Médicas, Facultad de Medicina, Universidad de los Andes, Bogotá, Colombia

**Keywords:** Chagas disease, T cell response, multifunctionality, inhibitory receptors, clonal exhaustion, immune activation

## Abstract

Chagas disease (ChD), a complex and persistent parasitosis caused by *Trypanosoma cruzi*, represents a natural model of chronic infection, in which some people exhibit cardiac or digestive complications that can result in death 20–40 years after the initial infection. Nonetheless, due to unknown mechanisms, some *T. cruzi*-infected individuals remain asymptomatic throughout their lives. Actually, no vaccine is available to prevent ChD, and treatments for chronic ChD patients are controversial. Chronically *T. cruzi*-infected individuals exhibit a deterioration of T cell function, an exhaustion state characterized by poor cytokine production and increased inhibitory receptor co-expression, suggesting that these changes are potentially related to ChD progression. Moreover, an effective anti-parasitic treatment appears to reverse this state and improve the T cell response. Taking into account these findings, the functionality state of T cells might provide a potential correlate of protection to detect individuals who will or will not develop the severe forms of ChD. Consequently, we investigated the T cell response, analyzed by flow cytometry with two multicolor immunofluorescence panels, to assess cytokines/cytotoxic molecules and the expression of inhibitory receptors, in a murine model of acute (10 and 30 days) and chronic (100 and 260 days) ChD, characterized by parasite persistence for up to 260 days post-infection and moderate inflammation of the colon and liver of *T. cruzi*-infected mice. Acute ChD induced a high antigen-specific multifunctional T cell response by producing IFN-γ, TNF-α, IL-2, granzyme B, and perforin; and a high frequency of T cells co-expressed 2B4, CD160, CTLA-4, and PD-1. In contrast, chronically infected mice with moderate inflammatory infiltrate in liver tissue exhibited monofunctional antigen-specific cells, high cytotoxic activity (granzyme B and perforin), and elevated levels of inhibitory receptors (predominantly CTLA-4 and PD-1) co-expressed on T cells. Taken together, these data support our previous results showing that similar to humans, the *T. cruzi* persistence in mice promotes the dysfunctionality of T cells, and these changes might correlate with ChD progression. Thus, these results constitute a model that will facilitate an in-depth search for immune markers and correlates of protection, as well as long-term studies of new immunotherapy strategies for ChD.

## Introduction

During the last decades, animal models have facilitated the study of complex diseases to analyze the components that potentially explain human pathologies. Indeed, studies using mice have provided major insights into the immunopathology of infectious diseases (1, 2). Protozoa are complex eukaryotic unicellular organisms because of their structure and metabolic activities (3). These parasites are a challenge to human health and a global threat because they persist for long periods of time, causing severe pathologies. Some species, such as *Leishmania* spp., *Plasmodium* spp., and *Trypanosoma cruzi*, are human pathogens, causing leishmaniasis, malaria, and Chagas disease (ChD), respectively. Although these illnesses were described many years ago, no vaccines are currently available and their effective etiological treatments are limited (4, 5). Thus, the use of appropriate animal models is crucial to understand the immunopathogenesis of these infectious diseases, to develop vaccines or immunotherapy strategies and to explore alternative approaches to treat infected individuals. A striking example of this success is the measurement of the quality (or multifunctional capacity) of the CD4^+^ T cell cytokine response as a crucial determinant for monitoring vaccine-mediated protection against *L. major* and *Plasmodium berghei* infection in mice (6, 7).

Furthermore, ChD, which is caused by the obligate intracellular protozoan *T. cruzi*, is a potentially life-threatening illness that was discovered more than 100 years ago and is responsible for more than seven times as many disability-adjusted life-years lost as malaria (8, 9). An estimated 6 to 7 million people have ChD (8), but it is increasingly spreading in non-endemic areas, mainly due to the migration of people from South and Central America (10). ChD, a complex and persistent parasitosis, has two clinical phases: acute and chronic. The acute phase lasts ~6–8 weeks and is usually asymptomatic in 90% of individuals or causes non-specific symptoms (11, 12). If infected individuals are not treated, the infection evolves into the chronic phase; ~60–70% of these patients will have the asymptomatic clinical form (indeterminate), while the remaining infected individuals develop the symptomatic clinical form (determinate) 20–40 years after the initial infection and exhibit cardiac, digestive, or mixed complications due to as yet unknown mechanisms (9). The etiological treatments in the chronic phase are controversial due to their toxicity and the lack of evidence for efficacy of the treatment. To date, the mechanisms involved in the pathogenesis of the disease are not well-established, and the immune mechanisms that prevent the progression of the chronic phase are being studied (9).

The long natural history of ChD makes the monitoring of patients and the sequential study of the immune mechanisms underlying protection or disease pathogenesis difficult. Indeed, experimental models of *T. cruzi* infection have been successfully used to propose or develop new strategies to combat ChD. To date, several animal models have been used to study many aspects of *T. cruzi* infection (13), including zebrafish (14), rabbits (15), dogs (16), rats (17), mice (18), and non-human primates (19). Given their small size and cost-effectiveness of laboratory maintenance, mice are one of the most promising animal models used to study this parasitic disease (18, 20).

Similar to other intracellular pathogens, *T. cruzi* induces CD4^+^ Th1 and CD8^+^ Tc1 cell responses, resulting in the secretion of cytokines and the release of cytotoxic granules upon antigen presentation (21, 22). Interestingly, in protozoan models of infection, the multifunctional response of T cells is essential for efficient parasite control (6). In contrast, in models of persistent infection, the failure to control the infection has been associated with the presence of T cells exhibiting a pronounced state of dysfunctionality known as T cell exhaustion, which is characterized by a monofunctional response, as measured by cytokine secretion, and increased inhibitory receptor co-expression on T cells (23, 24). Indeed, according to previous studies by our research group, T cells from individuals with advanced forms of ChD (i.e., established chagasic cardiomyopathy) have a higher monofunctional capacity and increased inhibitory receptor co-expression than T cells from asymptomatic patients with ChD (25, 26). Interestingly, when evaluating T cell responses in asymptomatic patients treated with anti-parasitic agents, a better quality or functional phenotype of T cells (i.e., increased proportion of multifunctional *T. cruzi*-specific CD8^+^ T cells) and decreased inhibitory receptor expression of 2B4 (CD244), CD160, TIM-3 (CD366), CTLA-4 (CD152), and PD-1 (CD279) on T cells have been observed (27). Based on these data, changes in these T cell immune parameters (such as multifunctionality and dysfunctionality) are potentially related to the failure of parasite control and ChD progression. However, a higher proportion of cytotoxic T cells, detected in chronically infected mice and in patients with symptomatic forms of ChD, has been related to a mechanism of tissue damage induced by *T. cruzi* infection (28–31).

In an effort to develop an animal model that will facilitate the identification of immune markers and correlates of protection, and, in the long term, new immunotherapy strategies for ChD, in the present study, we analyzed whether experimental acute (10 and 30 days) and chronic (100 and 260 days) ChD alters the CD4^+^ Th1 and CD8^+^ Tc1 cell multifunctional capacities and inhibitory receptor co-expression on T cells in a murine model with a reticulotropic Y strain of *T. cruzi*.

## Materials and Methods

### Ethics Statement

This study was performed in accordance with the ethical standards of the Institutional Animal Care and Use Committee (IACUC, approval FUA-007-14) from the Unidad de Biología Comparativa (UBA) at Pontificia Universidad Javeriana (PUJ, Bogotá, Colombia). All animal studies were conducted in accordance with the “Guide for the Care and Use of Laboratory Animals” from UBA-PUJ. The present study was described according to the Animal Research: Reporting *in vivo* Experiments (ARRIVE) criteria from the National Center for the Replacement, Refinement and Reduction of Animals in Research (NC3Rs) (32).

### Mice

Female inbred BALB/cAnNCr mice (6–8 weeks old) were purchased from Charles River Laboratories International, Inc. (Wilmington, MA, USA) and housed in specific pathogen-free (SPF) animal facilities at the UBA-PUJ. The BALB/c mouse strain was chosen to minimize variability in comparisons with previous studies (22, 33–35). The animals were housed in ventilated racks in an animal biosafety level 2 (ABSL-2) room under constant noise-free environmental conditions at a room temperature of 21 ± 1°C, a humidity of 50 ± 1%, an air exchange rate of 22.55 air changes/h, and a day–night rhythm of 12–12 h (light phase from 6 a.m. to 6 p.m.) in polycarbonate cages (4 or 5 animals/cage) with sterile soft wood shaving bedding, which was changed weekly. The mice received filtered water (changed weekly) and a standard mouse maintenance diet *ad libitum*. Stress and microbiological monitoring (behavioral and animal welfare analyses, as well as microbiological and serological testing) were performed according to IACUC guidelines.

### Parasites

*Trypanosoma cruzi* trypomastigotes from the Y strain (MHOM/BR/00/Y isolate; discrete typing unit (DTU) TcII) were obtained by culture passage on a monolayer of renal fibroblast-like cells (VERO cells, ATCC CCL-81, Manassas, VA, USA). Then, Y strain trypomastigotes were passaged in female inbred BALB/cAnNCr mice at least 3 times to increase their virulence. The parasite strain was chosen to minimize variability in comparisons with previous studies (33, 36, 37).

### Mouse Infection

BALB/c mice were randomly divided into 4 experimental groups (G1–G4, 5 mice per group) and infected with the parasite. All mice were simultaneously intraperitoneally injected with 10^5^ Y strain trypomastigotes in 100 μl of 1 × PBS under aseptic conditions and euthanized by CO_2_ inhalation at different time points after infection. G1, G2, G3, and G4 mice were euthanized at 10, 30, 100, and 260 days post-infection (dpi), respectively. In addition, another group of mice (G5) was injected with 100 μl of 1 × PBS under the same conditions and euthanized on the same dpi described above. Parasitemia was evaluated daily in 5 μl of tail venous blood by performing a direct microscopic observation of 50 fields during the first 10 days, and then every 2 days until day 40 dpi. Data are presented as the number of parasites per ml. The sample size was determined based on the average number of mice used in previous studies of *T. cruzi* infection in mice (33, 38–40). The time periods for acute (10 and 30 dpi) or chronic phases (100 and 260 dpi) were selected based on previous reports that evaluated immunological parameters in *T. cruzi*-infected mice (33, 41–43). Animal welfare indicators (score sheet) were recorded weekly for the first 30 days and then every month thereafter. Supplementary Figure 1 shows the experimental design of the present work.

### *T. cruzi* Soluble Antigens (Ags)

The *T. cruzi* soluble Ags (*Tc*SAs) were obtained using previously described methods, with some modifications (44–46). Briefly, *T. cruzi* trypomastigotes (Y strain) were obtained on a monolayer of VERO cells (44, 45), which were cultured in DMEM (Eurobio, Les Ulis, France) supplemented with 10% FBS, 2 mM L-glutamine, 100 U/ml penicillin, 100 μg/ml streptomycin, and 0.01 M HEPES (Eurobio; Les Ulis, France) at 37°C in a humidified atmosphere of 5% CO_2_. Amastigotes and trypomastigotes (1:1 ratio) were collected from the VERO cell culture supernatants at 96–120 h post-infection (44). Then, the parasites were washed twice with cold 1 × PBS (Eurobio) and resuspended at a density of 1 × 10^6^ parasites/μl in lysis buffer as previously reported (46). Parasites were incubated on ice for 30 min, and the supernatants containing *Tc*SAs were collected by centrifugation at 12,000 × *g* for 15 min at 4°C and stored at −80°C until use. The protein concentrations were determined using the Bradford assay, and the protein profiles were analyzed using SDS-PAGE followed by Coomassie blue staining (Gibco BRL; Grand Island, NY, USA).

### Monoclonal Antibodies for Staining

The following conjugated Abs were used for cell surface staining: CD3-PerCP-Cy5.5 (clone 17A2), CD4-Alexa Fluor 700 (clone GK1.5), CD8a-APC-H7 (clone 53–6.7), PD-1-APC (clone J43), 2B4-FITC (clone 2B4), and CD160-PE-CF594 (clone CNX46-3) (BD Biosciences; San Jose, CA, USA). The conjugated antibodies for intracellular staining included: IFN-γ-PE-CF594 (clone XMG1.2), IL-2-BV-421 (clone JES6-5H4), TNF-α-PE-Cy7 (clone MP6-XT22) (BD Biosciences), perforin-APC (clone eBioOMAK-D) and granzyme B-PE (clone NGZB) (Thermo Fisher Scientific, Waltham, MA, USA). The Fixable Aqua Dead Cell Stain viability marker (LIVE/DEAD) (Invitrogen; Eugene, OR, USA) was used to exclude the dead cells. All conjugated antibodies were titrated and evaluated as previously described (45).

### Analysis of the Cell Surface and Intracellular Cytokine Staining Using Flow Cytometry

One million spleen cells were stained with two multicolor panels to assess the cytokine/cytotoxic response associated with CD4^+^ Th1 and CD8^+^ Tc1 cells and inhibitory receptors on T cells. Cells were cultured for 3 h at 37°C in a humidified atmosphere containing 5% CO_2_ with Mock (as a negative control) and *Tc*SAs (1 μg/ml) in the presence of anti-CD28 (1 μg/ml, clone 37.51, BD Pharmingen) and then incubated in the presence of brefeldin A (1 μg/ml) and monensin (0.7 μg/ml) (BD Biosciences) for an additional 9 h to examine the cytokine/cytotoxic response by T cells. After incubation in 96-well round-bottom tissue culture plates, the cells were stained with the LIVE/DEAD marker followed by anti-CD3, anti-CD4, and anti-CD8 Abs for 30 min in the dark at 4°C and washed with staining buffer. Cells were fixed and permeabilized with Cytofix/Cytoperm buffer (BD Biosciences), incubated with anti-IFN-γ, anti-TNF-α, anti-IL-2, anti-granzyme B, and anti-perforin antibodies for 30 min in the dark at 4°C and then washed with 1 × Perm/Wash buffer (BD Biosciences).

In 96-well round-bottom tissue culture plates, the cells were stained with the viability marker, incubated with the anti-CD3, anti-CD4, anti-CD8, anti-2B4, anti-CD160, and anti-PD-1 antibodies for 30 min in the dark at 4°C and then washed with staining buffer to assess the expression of inhibitory receptors. Then, cells were fixed and permeabilized with Cytofix/Cytoperm buffer for 20 min at 4°C prior to staining with the anti-CTLA-4 antibody and then washed with 1 × Perm/Wash buffer. At least 100,000 events, gated on live CD3^+^ cells, were acquired on a FACS Aria II flow cytometer (BD Biosciences). Data were analyzed using FlowJo 9.3 (Tree Star; Ashland, OR, USA), Pestle 1.7 (National Institutes of Health (NIH), Bethesda, MD, USA), and SPICE 5.3 (NIH) software. Dead and doublet cells were excluded from the analysis (Supplementary Figure 2). A positive cytokine/cytotoxic response was defined as a frequency >0.05%, which was determined as the median frequency of a T cell response obtained from uninfected mice after stimulation with *Tc*SA plus 1 SD after background subtraction (cells from each mouse cultured with Mock).

### Parasite Detection and Quantification Using Conventional and Quantitative PCR

Cardiac blood, skeletal muscle from the posterior leg, heart, colon and liver tissue were collected for DNA extraction. The blood was placed in guanidine hydrochloride-EDTA and stored at 4°C, while the other tissues were stored in absolute ethanol at 4°C. Genomic DNA (gDNA) was extracted using a High Pure PCR template preparation kit according to the manufacturer's instructions (Roche, Mannheim, Germany). Afterwards, conventional PCR (cPCR) was performed with the CytB Uni fw 5′-TCATCMTGATGAAAYTTYGG-3′ and CytB Uni rev 5′-ACTGGYTGDCCBCCRATTCA-3′ primers, which amplify the cytochrome B gene of small mammalian species, as described previously (47), to assess DNA integrity and exclude the presence of inhibitors in the sample. For parasite detection, cPCRs were performed using gDNA from all tissues with the primers S35 5′-AAATAATGTACGGG(T/G)GAGATGCATGA-3′ and S36 5′-GGTTCGATTGGGGTTGGTGTAATATA-3′, based on the conserved regions of minicircles from *T*. *cruzi* kinetoplast DNA, and the primers TcH2AF 5′-CGAGCTCTTGCCCACACGGGTGCT-3′ and TcH2AR 5′-CCTCCAAGCAGCGGATAGTTCAGG-3′, based on *T*. *cruzi* satellite DNA which amplify a fragment present in the non-coding region of the histone H2A from *T. cruzi*, using previously described conditions (48, 49). Subsequently, quantitative PCR (qPCR) was performed with the Cruzi 1 5′-ASTCGGCTGATCGTTTTCGA-3′ and Cruzi 2 5′-AATTCCTCCAAGCAGCGGATA-3′ primers and the Cruzi 3 5′-6FAM-CACACACTGGACACCAA-BBQ-3′ probe, which amplify a 166-bp segment of *T. cruzi* satellite DNA (50). Each sample was analyzed in duplicate (with a coefficient of variation of <20%), and the parasite load was estimated based on a standard curve. The curve was constructed with different concentrations of *T. cruzi* Y strain gDNA mixed with 50 ng of gDNA from the heart or colon tissue of one uninfected mouse, ranging from 10^−1^ to 10^4^ parasite equivalents per 50 ng of DNA (Supplementary Figure 3), as described previously (51). Parasite loads below the limit of quantification (LOQ) were set to LOQ/2 (0.05 parasite equivalents per 50 ng of DNA), as previously described (52). Amplification was performed using the Applied Biosystems™ QuantStudio™ 3 Real-Time PCR System (Applied Biosystems, USA) with previously described qPCR conditions (53). For both PCR methods, the following controls were included: reaction (water added in the room containing the reaction mixture), gray (water added in the room where the sample was added to the reaction), negative (genomic DNA from one uninfected mouse), and positive (genomic DNA from *T. cruzi*).

### Histopathology

Paraffin-embedded tissues were stained with hematoxylin and eosin (H&E), and the following features were blinded and analyzed: presence of inflammation, type of cellular infiltration and pathological changes (e.g., necrosis). Histopathological scores were assigned as follows using previously described methods (54): absent, mild, moderate, or severe.

### Statistical Analysis

Statistical analyses were performed using the Mann–Whitney U test for two groups. Correlations between the multifunctional T cell response and inhibitory receptor co-expression on T cells were analyzed using Spearman's rank correlation coefficient. The tests were two-tailed, and statistical significance was achieved at *p* < 0.05. GraphPad Prism 6.0b for Mac OS X software (GraphPad, San Diego, CA) was used for statistical analyses. The multifunctional and co-expression pie charts were compared using 10,000 permutations calculated with SPICE version 5.3 software (55).

## Results

### The Presence of *T. cruzi* Is Associated With Inflammatory Infiltration in the Colon and Heart of Experimental ChD Model Mice

**Figure 1:**
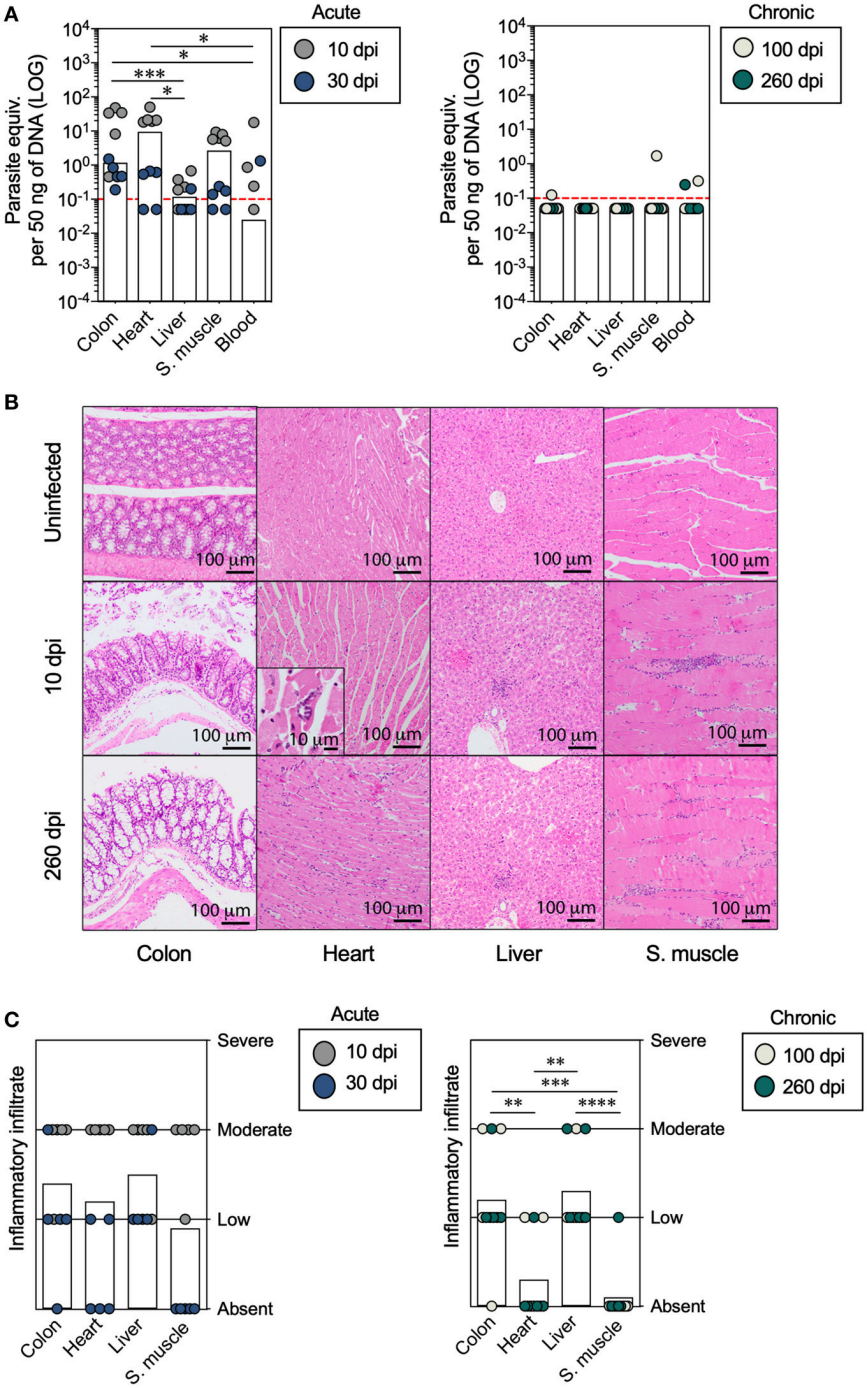
Analysis of the parasite load and inflammatory cell infiltration in mice with acute and chronic experimental ChD. **(A)** Parasite loads in the colon, heart, liver, skeletal muscle, and blood samples from mice with acute (left panel) and chronic (right panel) *T. cruzi* infections. The bar graphs show the median parasite equivalent per 50 ng of DNA (LOG_10_) in each tissue from each mouse. The dotted line represents the cut-off for the limit of detectable quantification (LOQ) based on serially diluted *T. cruzi*-spiked tissue DNA as described in the Materials and Methods (0.1 parasite equivalents per 50 ng of DNA). **(B)** Images of representative histopathological staining of cross-sections of tissues from mice with acute and chronic experimental ChD. **(C)** Inflammatory infiltrate scores in the colon, heart, liver, and skeletal muscle tissues from mice with acute (left panel) and chronic (right panel) *T. cruzi* infections. The bar graphs show the average inflammatory infiltrate scores in the tissues. The inflammatory infiltrate score was obtained as described in the Materials and Methods. *p < 0.05, **p < 0.01, ***p < 0.001, and ****p < 0.0001, Mann-Whitney *U*-test.

BALB/c mice were infected and followed for 260 dpi to experimentally mimic the course of a human *T. cruzi* infection (Supplementary Figure 1). After infection with 10^5^ Y strain trypomastigotes, parasitemia was observed at 3 dpi, peaked at 5 dpi [median (range), 9.3 × 10^4^ parasites/ml (3.1 × 10^4^-1.85 × 10^5^)], and became undetectable after 8 dpi (Supplementary Figure 4A). Then, we sought to compare the detection and quantification of the parasite in the colon, heart, liver, skeletal muscle, and blood of mice with acute (10 and 30 dpi) and chronic (100 and 260 dpi) experimental ChD. As expected, the parasite was detected in most of the tissues from acutely infected mice analyzed (colon: 90%, heart: 80%, liver: 30%, skeletal muscle: 60%, and blood: 100%), whereas it was found less often in tissues from chronically infected mice (colon: 20%, heart: 20%, liver: 10%, skeletal muscle: 10%, and blood: 20%) (Supplementary Table). Furthermore, mice with an acute infection showed higher parasite loads in the colon and heart than in the liver and blood. Although the parasite load was below the LOQ in the majority of chronically infected mice, when comparing the parasite loads according to the dpi, higher parasite loads in the colon, heart, liver, and skeletal muscle tissues were detected in mice at 10 or 30 dpi compared with those at 100 or 260 dpi (Supplementary Figure 4B). On the contrary, similar parasite loads in the colon, heart, liver, and skeletal muscle tissues were detected in mice at 100 and 260 dpi (Figure 1A).

Subsequently, we compared the histological findings in the colon, heart, liver, and skeletal muscle tissues of mice with acute and chronic experimental ChD. No changes in histology were observed in any of the tissues harvested from uninfected control mice, while mononuclear infiltration was present in all tissues (colon, heart, liver, and skeletal muscle) obtained from mice with acute and chronic experimental ChD (Figures 1B,C). The average inflammatory infiltrate was higher in the colon and liver tissues than in the heart and skeletal muscle tissues from all mice, and was more evident in tissues from the chronically infected mice (Figure 1C). Necrosis was mainly observed in the heart and skeletal muscle of mice with an acute infection (data not shown). Finally, the parasite detected using cPCR and qPCR was associated with inflammatory infiltration in the colon [*p* = 0.0281, OR (95% CI) = 14.0 (1.2–156.7)] and heart [*p* = 0.0325, OR (95% CI) = 21.0 (0.9–454.3)] of all infected mice (Supplementary Figure 5A), and higher parasite loads were observed in colon (*p* = 0.0201), heart (*p* = 0.0001), liver (*p* = 0.0071), and skeletal muscle (*p* = 0.0017) tissues with moderate inflammatory infiltrate compared with those with low or absent inflammatory infiltrate from *T. cruzi*-infected mice (Supplementary Figure 5B). Thus, the parasite persists for up to 260 dpi, preferably in liver, and high parasite loads induce a moderate inflammatory infiltrate in the colon, heart, liver, and skeletal muscle tissues from *T. cruzi*-infected mice.

### Acute *T. cruzi* Infection Induces Multifunctional T Cells, Whereas Chronic Infection Promotes Monofunctional T Cell Responses

**Figure 2:**
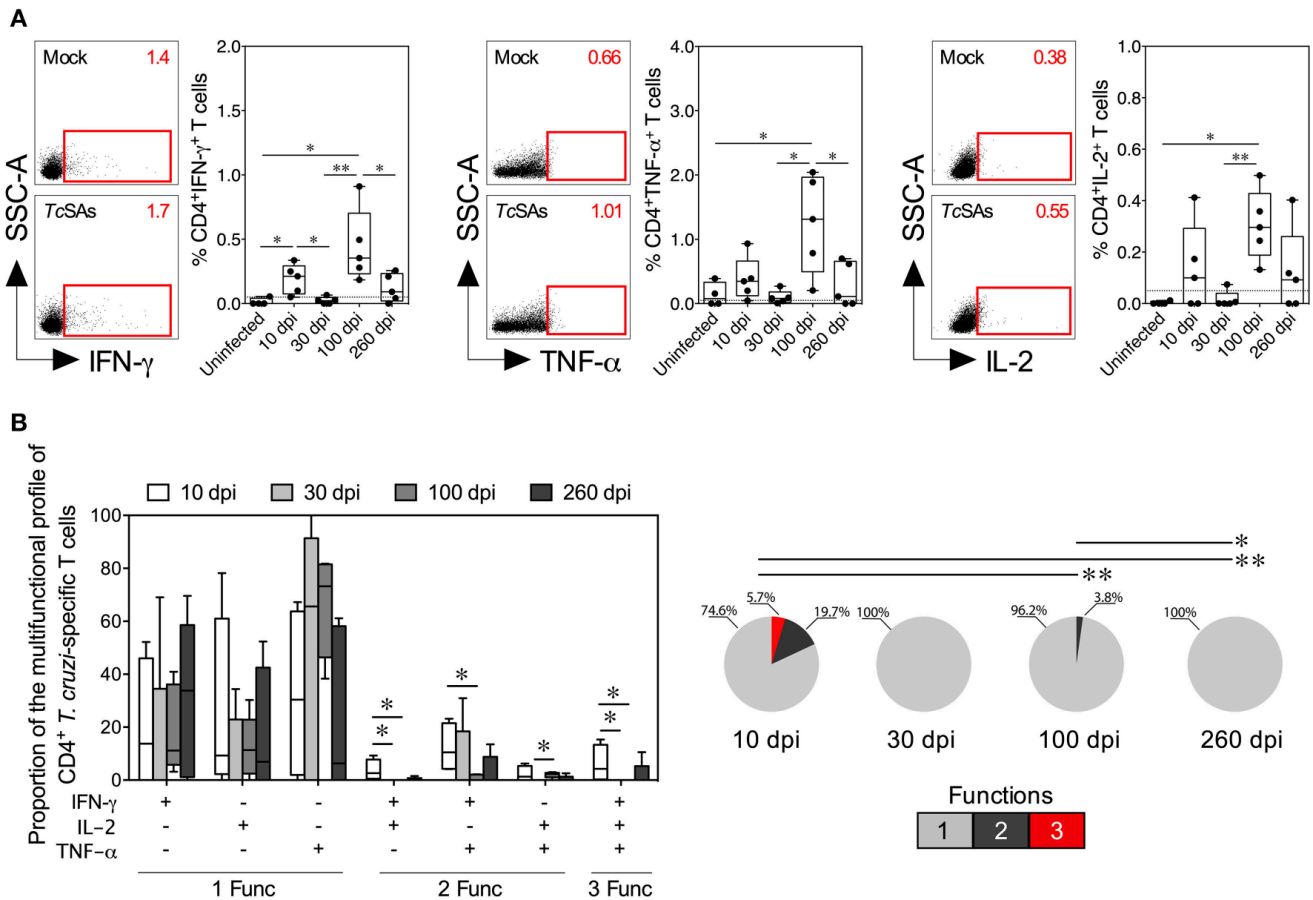
Functional activity profiles of *T. cruzi*–specific CD4^+^ Th1 cells from mice with acute and chronic experimental ChD. **(A)** Representative dot plot (left panel) and percentages (right panel) of CD4^+^ T cells producing IFN-γ, TNF-α, or IL-2 in mice with acute and chronic experimental ChD. The gates applied for the identification of cytokine production on the total population of CD4^+^ T cells were defined according to the cells cultured with Mock for each mouse. The number on the upper right side corresponds to the frequency detected in spleen cells cultured with Mock or *Tc*SAs. The dotted line represents the cut-off for the assessment of a positive cytokine response, as described in the Materials and Methods. **(B)** Proportions of the functional profiles of CD4^+^ T cells with one, two, or three functions after stimulation with *Tc*SA. The boxes (25–75th percentiles) and whiskers (minimum to maximum) show the median percentages **(A)** or proportions **(B)** of Ag-specific CD4^+^ T cells. The pie chart depicts the median proportion of Ag-specific CD4^+^ T cells, and the color depicts the T cells with one, two, or three functions. *p < 0.05 and **p < 0.01, Mann-Whitney *U*-test (boxes and whiskers) or permutation test (pie charts).

**Figure 3:**
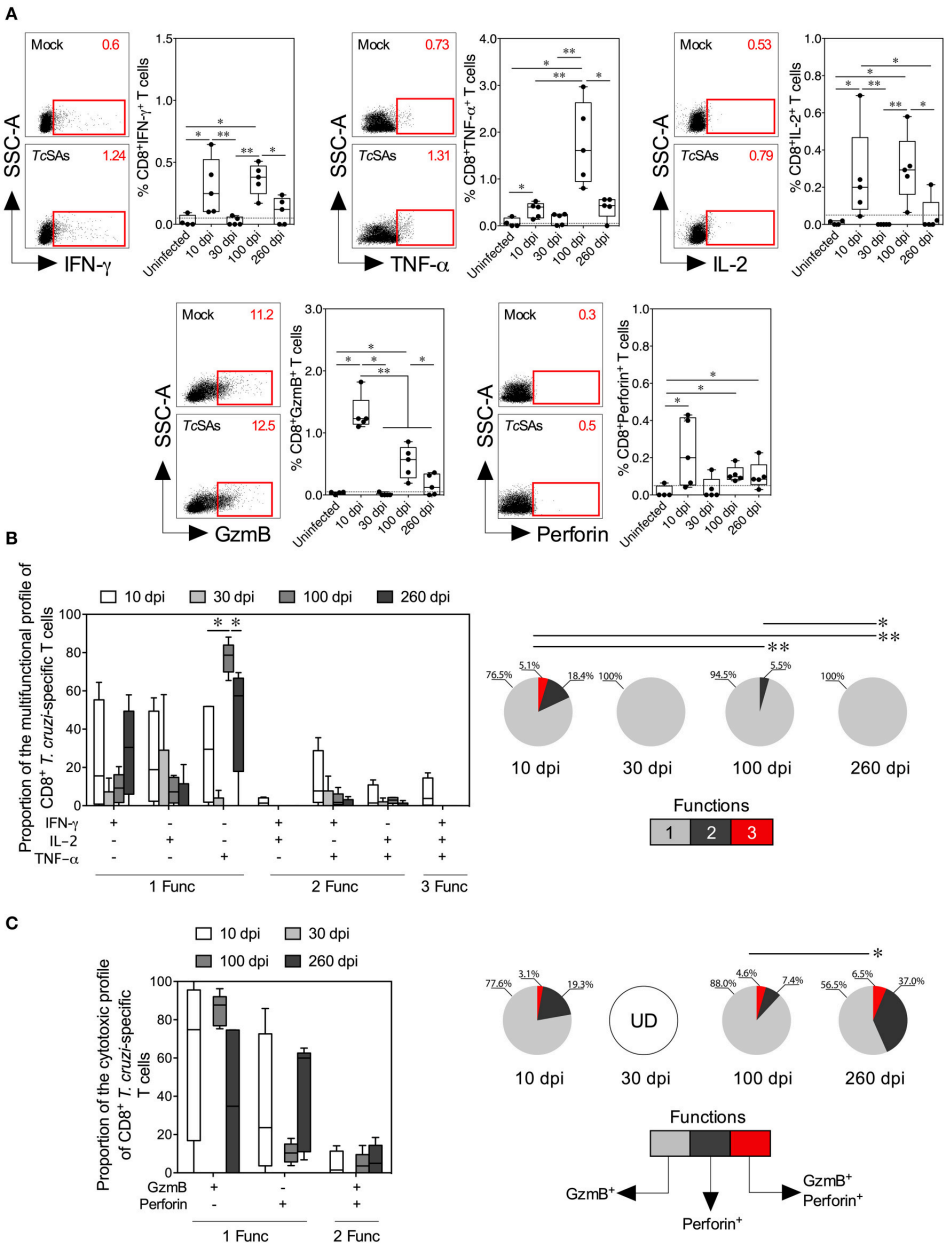
Functional activity profiles of *T. cruzi*–specific CD8^+^ Tc1 cells from mice with acute and chronic experimental ChD. **(A)** Representative dot plot (left panel) and percentages (right panel) of CD8^+^ T cells producing IFN-γ, TNF-α, IL-2, granzyme B, or perforin in mice with acute and chronic experimental ChD. The gates applied for the identification of cytokine, granzyme B or perforin production on the total population of CD8^+^ T cells were defined according to the cells cultured with Mock for each mouse. The number on the upper right side corresponds to the frequency of molecules detected in spleen cells cultured with Mock or *Tc*SAs. The dotted line represents the cut-off for the assessment of a positive response, as described in the Materials and Methods. **(B)** Proportions of the functional profiles of CD8^+^ T cells with one, two, or three functions after stimulation with *Tc*SA. **(C)** Proportions of the cytotoxic profiles of CD8^+^ T cells producing granzyme B or perforin from mice with acute and chronic experimental ChD stimulated with *Tc*SA. The boxes (25–75th percentiles) and whiskers (minimum to maximum) show the median percentages **(A)** or proportions **(B,C)** of Ag-specific CD8^+^ T cells. The pie chart depicts the median proportion of Ag-specific CD8^+^ T cells, and the color depicts the T cells with one, two, or three functions **(B)** or the production of granzyme B, perforin, or both **(C)**. *p < 0.05 and **p < 0.01, Mann-Whitney *U*-test (boxes and whiskers) or permutation test (pie charts). GzmB, granzyme B; UD, undetected.

The induction of a CD4^+^ Th1 and CD8^+^ Tc1 cell responses by the production of IFN-γ, TNF-α, IL-2, granzyme B, or perforin by T cells in response to *Tc*SA was evaluated as described in the Materials and Methods section to compare the function of T cells between acute and chronic *T. cruzi*-infected mice. Initially, the secretion of individual cytokines from T cells was assessed, and greater percentages of CD4^+^ and CD8^+^ T cells producing IFN-γ, TNF-α, or IL-2 were observed in infected mice at 10 and 100 dpi than in mice at 30 dpi. Comparable proportions of CD4^+^ T cells producing IFN-γ, TNF-α, or IL-2 and of CD8^+^ T cells producing IFN-γ or TNF-α were found in infected mice at 10 and 260 dpi (Figures 2A, 3A). Interestingly, in mice at 100 dpi, higher proportions of CD4^+^ and CD8^+^ T cells producing TNF-α appeared to be present compared with CD4^+^ and CD8^+^ T cells producing IFN-γ and IL-2 (Figures 2A, 3A). In CD8^+^ T cells, most mice at 260 dpi had low or undetectable levels of IL-2 (Figure 3A). When comparing the percentages of total cytokine-producing *T. cruzi*-specific T cells, as expected, increased percentages of Ag-specific CD4^+^ and CD8^+^ T cells were observed in mice at 10 (median: 0.6419 and 0.9386%, respectively) and 100 dpi (median: 1.067 and 1.488%, respectively) compared with those at 30 (median: 0.2731 and 0.1000%, respectively) and 260 dpi (median: 0.4743 and 0.8312%, respectively). Notably, although lower percentages of T cells producing IFN-γ, TNF-α, or IL-2 were observed in mice at 30 dpi, the total Ag-specific responses of CD4^+^ and CD8^+^ T cells were higher in these mice (median: 0.0671 and 0.0876%, respectively) than in uninfected mice (data not shown). When individual cytotoxic molecules on CD8^+^ T cells were assessed, greater percentages of T cells expressing granzyme B or perforin were observed in infected mice at 10 dpi than in the other experimental groups (Figure 3A).

Next, a Boolean gating approach was used to compare the proportions of multifunctional and monofunctional cells with CD4^+^ Th1 and CD8^+^ Tc1 cell profiles among mice at 10, 30, 100, and 260 dpi. At 10 dpi, mice displayed higher proportions of multifunctional CD4^+^ and CD8^+^ T cells with two and three functions than at 30, 100 or 260 dpi. At 100 dpi, CD4^+^ and CD8^+^ T cells had two functions in infected mice, whereas T cells collected from mice at 30 and 260 dpi showed one function. Interestingly, the predominant functional profiles of Ag-specific CD4^+^ and CD8^+^ T cells were similar in all mice, i.e., preferentially, T cells with one function produced TNF-α, whereas those with two functions produced IFN-γ and TNF-α. Multifunctional CD4^+^ T cells producing IFN-γ and IL-2, IFN-γ and TNF-α, and IL-2 and TNF-α occurred more frequently in mice at 10 dpi than at 100 dpi, whereas monofunctional CD8^+^ T cells producing TNF-α were more commonly observed in mice at 100 dpi than at 10 and 260 dpi (Figures 2B, 3B). We next compared the proportions of cytotoxic profiles of CD8^+^ T cells by measuring granzyme B and perforin production in mice at 10, 30, 100, and 260 dpi. Notably, no CD8^+^ T cells producing granzyme B or perforin were detected in mice at 30 dpi. An increased proportion of CD8^+^ T cells producing granzyme B and perforin was detected in mice at 260 compared with that at 100 dpi (Figure 3C).

### Inhibitory Receptors Are Upregulated During Acute and Chronic Experimental ChD

**Figure 4:**
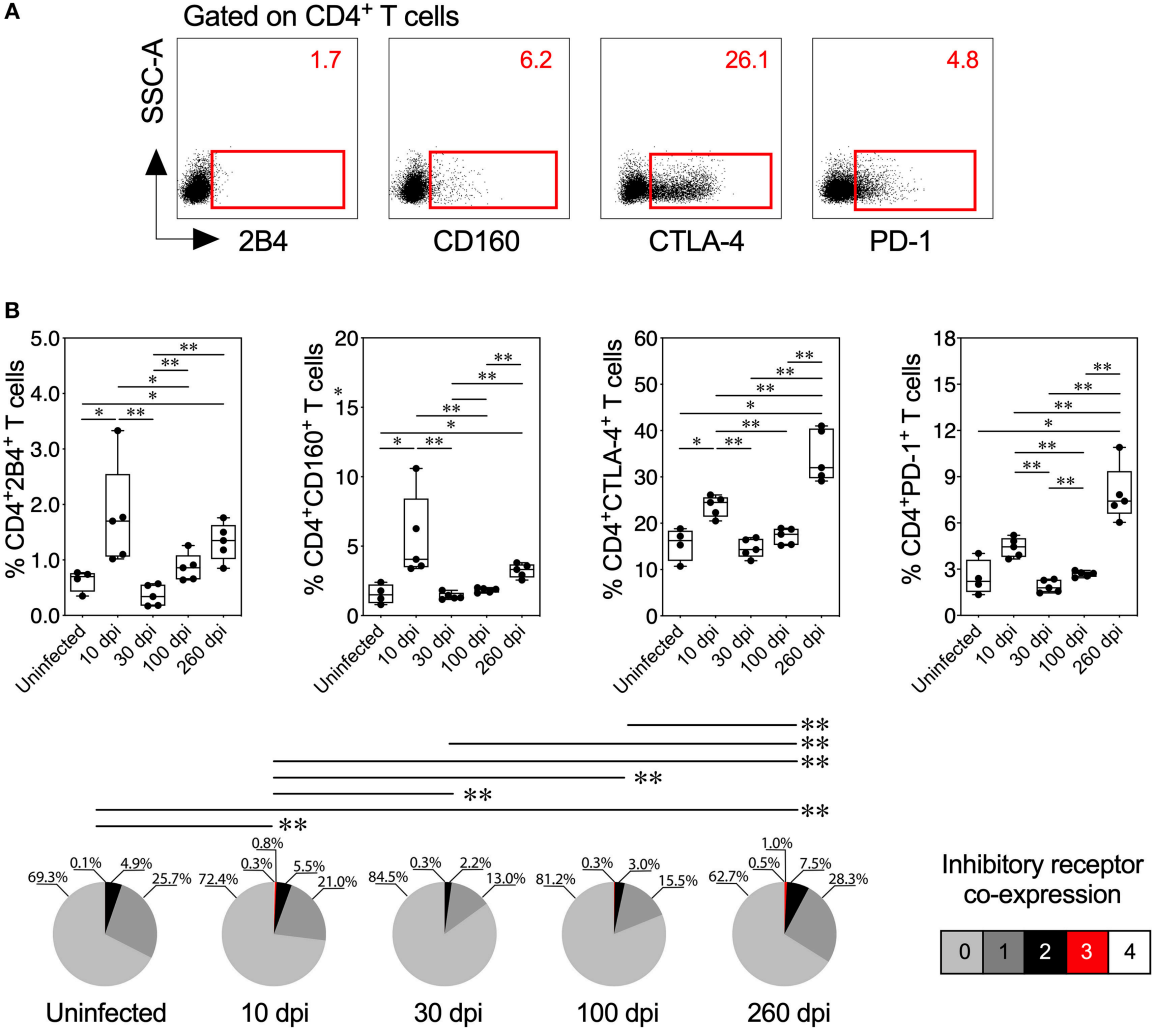
Inhibitory receptor expression and co-expression on CD4^+^ T cells from mice with acute and chronic experimental ChD. **(A)** Representative dot plot of the gating strategy for CD4^+^ T cell expressing 2B4, CD160, CTLA-4, or PD-1. The number on the upper right side corresponds to the frequency of molecules detected in CD4^+^ T cells. **(B)** Percentages (top panel) and median proportions (bottom panel) of CD4^+^ T cells expressing 2B4, CD160, CTLA-4, or PD-1 in mice with experimental ChD. The boxes (25–75th percentiles) and whiskers (minimum to maximum) show the median percentages and range of expression of inhibitory receptors on CD4^+^ T cells. The pie chart depicts the median proportion of inhibitory receptors. *p < 0.05 and **p < 0.01, Mann-Whitney *U-*test (boxes and whiskers) or permutation test (pie charts).

**Figure 5:**
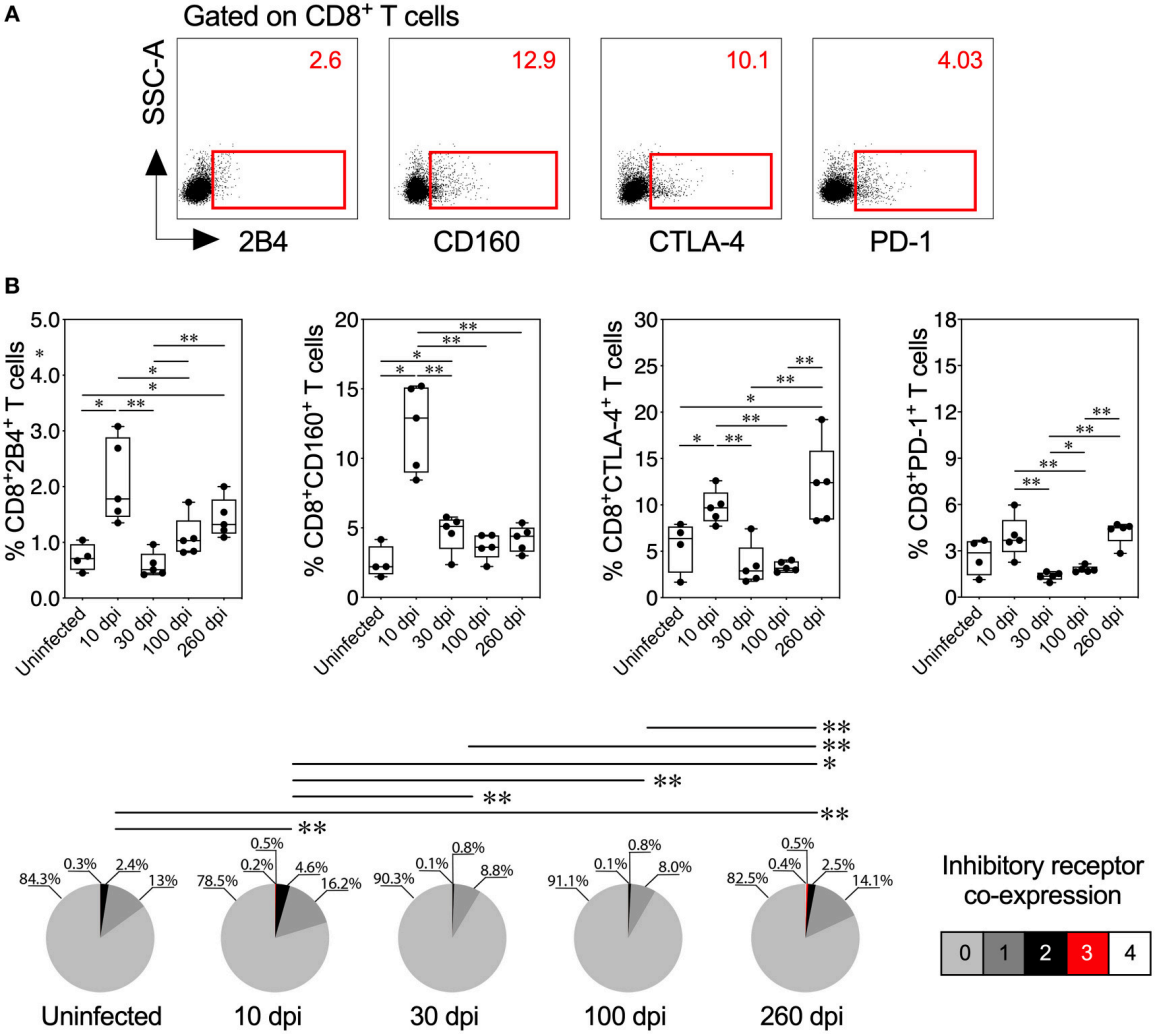
Inhibitory receptor expression and co-expression on CD8^+^ T cells from mice with acute and chronic experimental ChD. **(A)** Representative dot plot of the gating strategy for CD8^+^ T cell expressing 2B4, CD160, CTLA-4, or PD-1. The number on the upper right side corresponds to the frequency of molecules detected in CD8^+^ T cells. **(B)** histopathological Percentages (top panel) and median proportions (bottom panel) of CD8^+^ T cells expressing 2B4, CD160, CTLA-4, or PD-1 in mice with experimental ChD. The boxes (25–75th percentiles) and whiskers (minimum to maximum) show the median percentages and range of expression of inhibitory receptors on CD8^+^ T cells. The pie chart depicts the median proportion of inhibitory receptors. *p < 0.05 and **p < 0.01, Mann-Whitney *U*-test (boxes and whiskers) or permutation test (pie charts).

Because inhibitory receptors have been classically characterized as molecules that regulate or inhibit the activation of T cells during acute and chronic infection, respectively (23, 56), we compared the inhibitory receptor expression or co-expression on T cells from mice with acute and chronic experimental ChD (Figures 4A, 5A). Increased expression of some inhibitory receptors was observed on CD4^+^ and CD8^+^ T cells from mice acutely and chronically infected with *T. cruzi*. Interestingly, 2B4, CD160, CTLA-4, or PD-1 were expressed at higher levels on CD4^+^ and CD8^+^ T cells from infected mice at 10 dpi than in uninfected mice and infected mice at 30 or 100 dpi, suggesting that the acute *T. cruzi* infection induced the expression of these molecules. Indeed, similar levels of 2B4, CD160, CTLA-4, or PD-1 expression were observed on T cells from uninfected mice and infected mice at 30 dpi, and in some cases, infected mice at 100 dpi (Top panel, Figures 4B, 5B). Additionally, CD4^+^ and CD8^+^ T cells obtained from infected mice at 100 dpi expressed 2B4 or PD-1 at higher levels than T cells from infected mice at 30 dpi, and mice at 260 dpi exhibited higher expression of CTLA-4 or PD-1 than mice at 100 dpi. Interestingly, chronically infected mice (at 260 dpi) exhibited increased or similar expression of CTLA-4 or PD-1 on CD4^+^ and CD8^+^ T cells compared with infected mice at 10 dpi (Top panel, Figures 4B, 5B). Upon analyzing the co-expression of these inhibitory molecules using a Boolean gating approach, the most prevalent population observed consisted of CD4^+^ and CD8^+^ T cells expressing CTLA-4 (median: 15.35 and 3.4%, respectively), CTLA-4 and PD-1 (median: 2.08 and 0.44%, respectively), and CD160, CTLA-4, and PD-1 (median: 0.05 and 0.06%, respectively) (data not shown). Notably, CD4^+^ and CD8^+^ T cells that co-expressed 2B4, CD160, CTLA-4, and PD-1 were detected in mice at 10 (median: 0.3 and 0.2%, respectively) and 260 dpi (median: 0.5 and 0.4%, respectively) but not in mice at 30 and 100 dpi (Bottom panel, Figures 4B, 5B). Thus, 2B4, CD160, CTLA-4, and PD-1 were transiently expressed on T cells after *T. cruzi* infection but were then rapidly downregulated. However, the expression of these molecules increased again in chronically *T. cruzi*-infected mice, and high levels of co-expression were maintained up to 260 dpi.

We correlated the Ag-specific T cells endowed with two and three functions and T cells that co-expressed three and four inhibitory receptors in mice with acute and chronic experimental ChD to determine whether multifunctionality was associated with the co-expression of inhibitory receptors in experimental ChD. In acutely infected mice (10 and 30 dpi), the percentage of multifunctional CD4^+^ and CD8^+^ T cells correlated positively with the level of inhibitory receptor co-expression on CD4^+^ and CD8^+^ T cells (Spearman's r = 0.8896, *p* = 0.0013 and Spearman's r = 0.8211, *p* = 0.0067, respectively). In contrast, in chronically infected mice, the percentage of multifunctional CD8^+^ T cells correlated negatively with the level of inhibitory receptor co-expression on CD8^+^ T cells (Spearman's *r* = −0.8232, *p* = 0.0046), but no correlation was observed with CD4^+^ T cells from chronically infected mice (data not shown). In addition, a positive correlation between the percentage of multifunctional T cells and 2B4 and CD160 expression on CD4^+^ and CD8^+^ T cells was observed in acutely infected mice, and a negative correlation was observed between the percentage of multifunctional CD8^+^ T cells and CTLA-4 and PD-1 expression on CD8^+^ T cells in chronically infected mice (Supplementary Figure 6).

**Figure 6:**
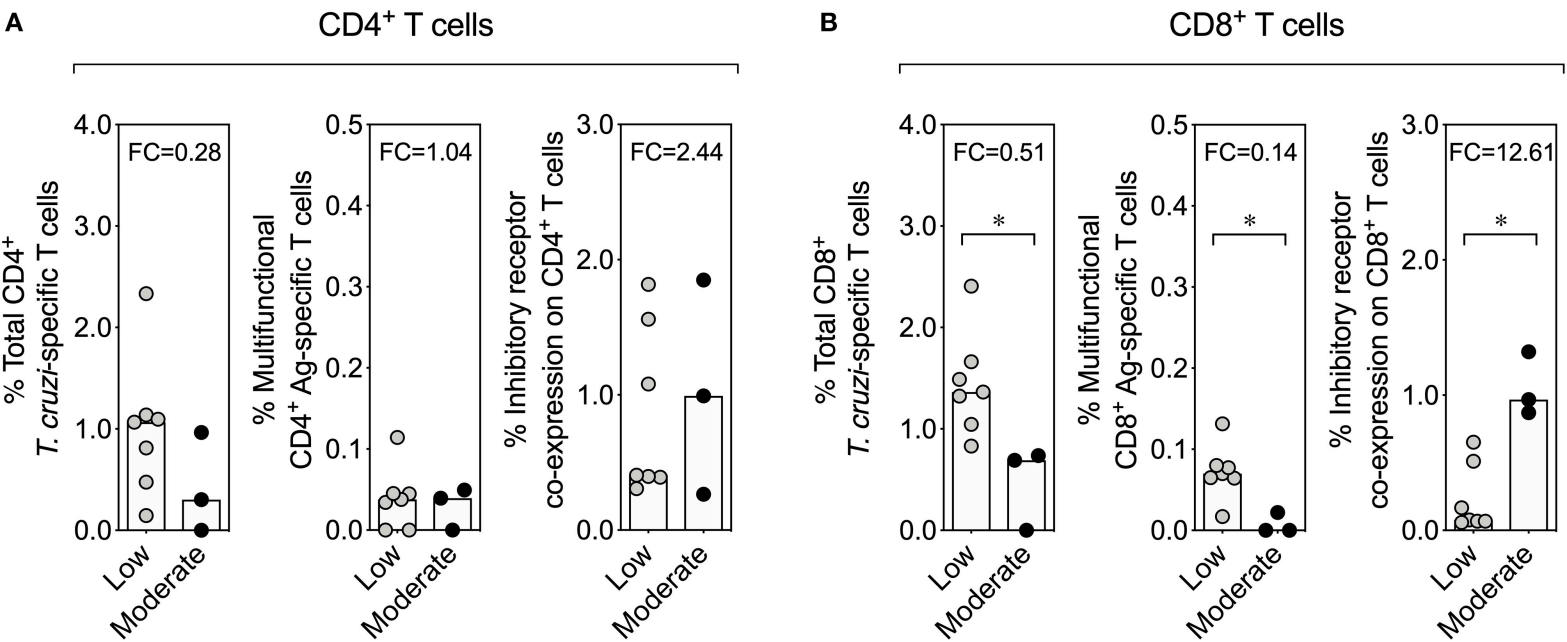
Effector function of T cells and inhibitory receptor co-expression on T cells according to the inflammatory infiltrate score in the liver tissue in chronically *T. cruzi*-infected mice. Comparison of the effector function of T cells and the inhibitory receptor co-expression on T cells between chronically *T. cruzi*-infected mice with moderate and low inflammatory infiltrate in the liver tissue. The comparative analysis with CD4^+^ and CD8^+^ T cells is shown in **(A,B)**, respectively. The bar graphs show the median percentages of total cytokine-producing *T. cruzi*-specific T cells, percentages of multifunctional T cells endowed with two and three functions, or percentages of T cells co-expressing three and four inhibitory receptors on T cells. *p < 0.05, Mann-Whitney *U*-test. FC, fold change.

Finally, given that moderate inflammatory infiltrate and high parasite load in liver in chronically infected mice at 260 dpi could be related with the fact that the reticulotropic Y strain of *T. cruzi* causes liver pathology (Supplementary Figure 5B) (57, 58), we analyzed whether there was any relationship between the inflammatory infiltrate in liver tissue and the functional activity or inhibitory receptor co-expression in chronically *T. cruzi*-infected mice. In 3 chronically infected mice at 100 and 260 dpi was found a moderate inflammatory infiltrate in the liver tissue, whereas in the rest of the mice (7 mice) it was detected a low inflammatory infiltrate. The comparison of the effector function and the inhibitory receptor expression revealed a reduction of antigen-specific multifunctional CD8^+^ T cells and an increase in the inhibitory receptor co-expression on CD8^+^ T cells in chronically infected mice that presented moderate inflammatory infiltrate in the liver tissue than in mice with low inflammatory infiltrate. However, no differences were found in effector function and the inhibitory receptor co-expression on CD4^+^ T cells in chronically infected mice that presented moderate or low inflammatory infiltrate in the liver tissue (Figure 6). In summary, the pathology in liver tissue in chronically *T. cruzi*-infected mice could be related with the dysfunctionality of CD8^+^ T cells, characterized by a low multifunctional effector T cell response and a high expression of inhibitory receptors on T cells.

**Figure 7:**
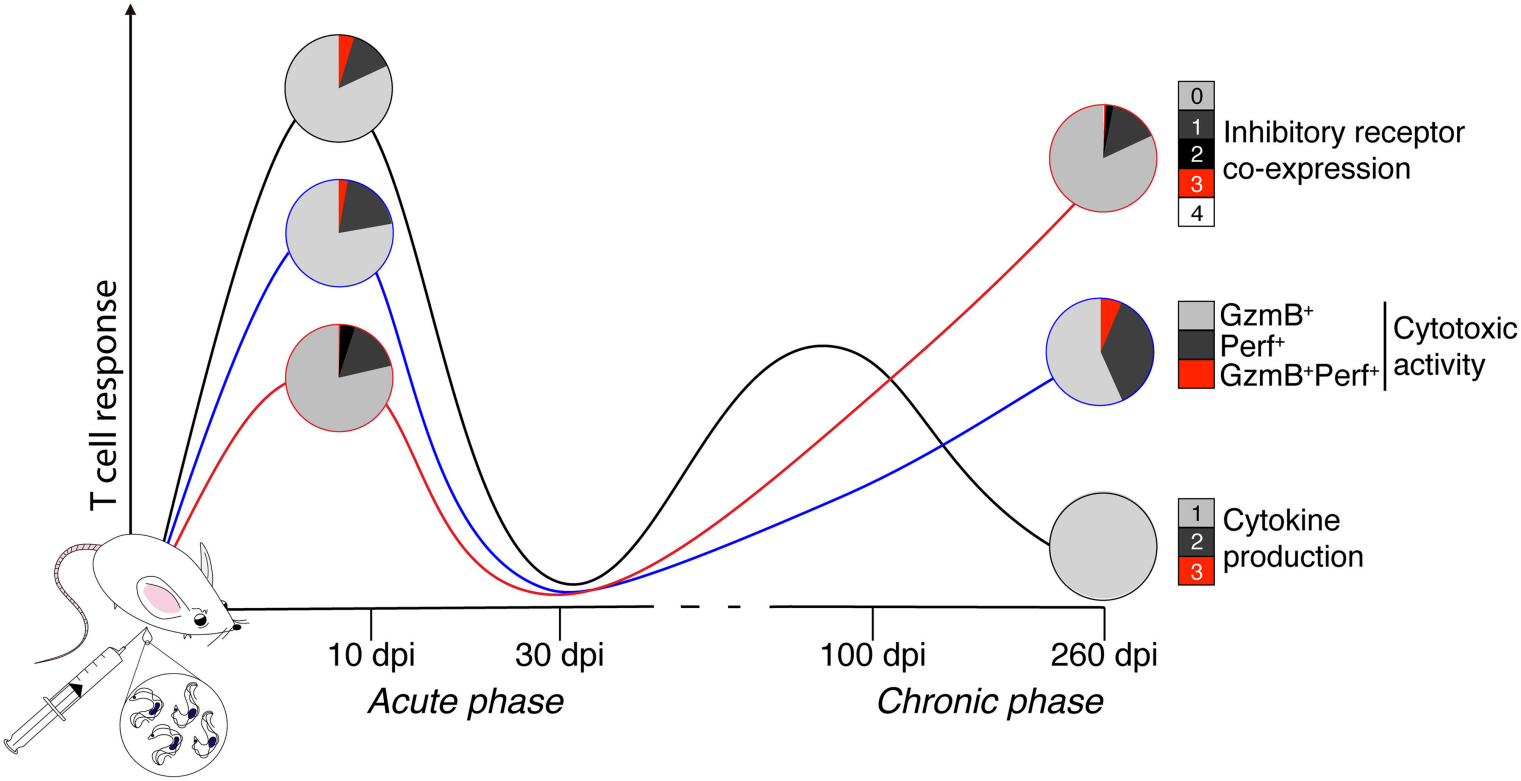
Schematic depicting the multifunctionality and dysfunctionality of T cells in an animal model of acute and chronic Chagas disease. In the present study, whether experimental acute (10 and 30 days) and chronic (100 and 260 days) ChD alters the CD4^+^ Th1 and CD8^+^ Tc1 cell multifunctional capacities and inhibitory receptor co-expression on T cells was analyzed in a murine model. Representative results of CD8^+^ T cells across the experimental infection conditions with *T. cruzi* are shown. The black, blue, and red lines show the median proportions of functional and cytotoxic activity and the co-expression of inhibitory receptors, respectively. The colors in the pie charts depict the number of T cell functions, as well as the inhibitory receptors co-expressed on CD8^+^ T cells in the acute and chronic phases of experimental ChD. Based on our results, an acute *T. cruzi* infection induces a multifunctional T cell response and high inhibitory receptor co-expression on T cells, whereas chronic *T. cruzi* infection is characterized by a monofunctional T cell response, high cytotoxic activity, and high levels of inhibitory receptor co-expression. GzmB, granzyme B; Perf, perforin.

Based on our results, an acute *T. cruzi* infection with the reticulotropic Y strain induces a multifunctional CD4^+^ Th1 and CD8^+^ Tc1 cell responses and high inhibitory receptor co-expression on T cells (2B4 and CD160), while a chronic *T. cruzi* infection is characterized by a monofunctional T cell response, high cytotoxic activity, and high levels of inhibitory receptor co-expression (CTLA-4 and PD-1) (Figure 7).

## Discussion

The acquired T cell dysfunction, known as T cell exhaustion, is a state commonly observed during chronic infections and cancer, and is associated with the severity of infection or pathology. This phenomenon is characterized by the gradual loss of antigen-specific T cell effector capacity and increased inhibitory receptor expression and co-expression on CD4^+^ and CD8^+^ T cells (23, 24, 59). In ChD, chronically infected individuals have dysfunctional CD8^+^ T cells exhibiting impaired cytokine production and increased inhibitory receptor co-expression, similar to the findings described above (25, 26). Interestingly, an anti-parasitic treatment improves the response of antigen-specific CD8^+^ T cells and decreases inhibitory receptor co-expression (27), suggesting that, similar to other models, changes in these T cell immune parameters are potentially related to the failure of the etiological treatment and progression of ChD. Thus, in the present study, we analyzed whether acute and chronic experimental ChD alters the multifunctional capacity and inhibitory receptor co-expression on T cells. In summary, acute experimental ChD in BALB/c mice induced a multifunctional CD4^+^ Th1 and CD8^+^ Tc1 cell responses and high inhibitory receptor co-expression on T cells. In contrast, chronically infected mice presented monofunctional antigen-specific T cells, high cytotoxic activity, and high levels of inhibitory receptor co-expression on T cells. In addition, we observed that the pathology in liver tissue in chronically *T. cruzi*-infected mice could be related with the dysfunctionality of CD8^+^ T cells, characterized by a low multifunctional effector T cell responses and high inhibitory receptor expressions on T cells. To our knowledge, this study is the first to provide evidence suggesting that similar to humans, chronic *T. cruzi* infection in mice leads to T cell exhaustion with impaired cytokine production and increased inhibitory receptor co-expression.

To date, experimental models of ChD have shown substantial variability in terms of infection outcomes, parasite load and tropism that depend on both the host and the parasite (60, 61). For instance, C57BL/6 mice are more resistant to *T. cruzi* infection than BALB/c mice, which show intermediate resistance to parasite infection (33, 61). In addition, *T. cruzi* is a parasite that includes different genetic lineages, known as DTUs (62), which have shown different tropisms (e.g., of the reticulotropic or myotropic strains) and infection outcomes in experimental models of ChD that causes heart, gastrointestinal tract, or liver pathologies (48, 60). For example, a lower parasite load has been observed in chronic *T. cruzi*-infected mice than in acutely infected mice (63), but these results can vary, even in studies using the same parasite strain (64). Indeed, another parameter that can affect the evolution of ChD is the number of parasites infecting the host, because murine models have shown that a high inoculum correlates with parasite persistence, inflammation or tissue damage (65, 66). Given this feature and to control the effect of the inoculum in our experimental ChD model, in a pilot experiment, we infected mice with different inocula (10^4^ and 10^5^ parasites) to select a dose that would allow for the detection of high levels of parasitemia, as shown here. However, it is still necessary to understand how the inoculum affects the course of the *T. cruzi*-infected humans, as the experimental dose of parasites may not reflect what occurs naturally. Thus, given the complexity of *T. cruzi* infection (60), the development of models that have similar characteristics to humans is important and potentially useful for identifying immune markers and correlates of protection, and, in the long term, new immunotherapy strategies for ChD. Studies using acute experimental models of ChD have shown that *T. cruzi* infection leads to transient mononuclear inflammatory infiltrates in many tissues, including the colon, heart, liver, and skeletal muscle, which decrease during chronic infection (54). Here, *T. cruzi* infection induced a mononuclear inflammatory infiltrate that was potentially related to the presence of the parasite in tissues, as it was observed in nearly all of the colon and heart tissue samples obtained from infected mice. Additionally, high parasite loads were observed in colon, heart, liver, and skeletal muscle tissues with moderate inflammatory infiltrate obtained from mice with acute and chronic infections compared with those with low or absent inflammatory infiltrate, suggesting that our experimental model is similar to that of other studies (54, 67). As it was to be expected, reticulotropic strains, such as Y strain, might be involved in increased inflammatory infiltrates in liver and spleen tissues and causes pathology as reported previously (57, 58), but to evaluate the pathological forms of experimental ChD, is important to implement other methodological approaches, such as the measurement of heart rate or the identification of fibrosis in tissue samples (57, 60, 68). However, to date, it has been very difficult to establish the direct relationship among the parasite load, the inflammatory infiltrate and the pathogenesis of ChD as previously described (69).

During acute infection, naïve antigen-specific T cells are activated, proliferate, acquire effector functions, and differentiate into effector T cells that are capable of mounting an immune response that will control or eliminate the pathogen (70). In contrast, during a chronic infection, the panorama is more complex; although antigen-specific effector T cells are developed, these T cell subsets lose their effector functions hierarchically. IL-2 production and cytotoxic capacity are the first functions to be compromised, followed by TNF-α production, and finally, IFN-γ production (23, 24). Additionally, T cells with this dysfunctional phenotype express inhibitory molecules such as PD-1 and CTLA-4 at high levels (71). This exhaustion state has been reported in infections caused by other parasites, such as *Plasmodium* spp. (72, 73), *Toxoplasma gondii* (74, 75), and *Leishmania* spp. (76–78), in human and mouse models. Although the precise features of T cell exhaustion vary during infection (24), a partial (i.e., poor effector response) or completely (i.e., a lack of effector capacity) dysfunctional T cell effector response can occur (23, 79). During a chronic *T. cruzi* infection, patients with advanced-stage ChD disease exhibit a lower percentage of T cells producing IFN-γ, TNF-α, or IL-2 and a lower proportion of multifunctional T cells than patients in the early stage of the disease (25), as well as decreased proliferation (59), which is associated with a decrease in the number T stem cell memory cells (26). Indeed, T cells from *T. cruzi*-infected children are more multifunctional than T cells from *T. cruzi*-infected adults, which have monofunctional T cells, suggesting that a persistent parasite infection promotes the exhaustion of T cells and contributes to the long-term progression of the disease in infected individuals (80). Here, as expected, acutely (10 dpi) infected mice showed an increased proportion of multifunctional T cells that produced cytokines and cytotoxic activity compared to infected mice at 30 dpi or chronically infected mice (100 or 260 dpi), as previously shown (81); however, although the elimination of the parasite was not achieved, a reduction in the parasite load in the colon, heart, liver, skeletal muscle, and blood was detected in chronically *T. cruzi*-infected mice. These chronically infected mice at 100 dpi showed a higher percentage of T cells producing IFN-γ, TNF-α, or IL-2 and a higher proportion of multifunctional T cells endowed with two functions than mice chronically infected at 260 dpi. Thus, chronic *T. cruzi* infection in mice potentially leads to partial T cell exhaustion or poor T cell effector functions, as has been observed in adult patients with ChD at advanced or severe disease stages (25). Interestingly, in our experimental ChD model that included the reticulotropic Y strain of the parasite allowed us to reveal that chronically infected mice with moderate inflammatory infiltrate in liver tissue has low percentages of antigen-specific multifunctional T cells, suggesting that a poor effector T cell response might be related with the liver pathology. However, validation of the T cell response in chronically infected mice with a myotropic *T. cruzi* strain is still necessary to validate our results. Under our experimental conditions, no direct relationship between the parasite load in tissues and the effector response of the CD4^+^ and CD8^+^ T cells was found. We hypothesized that this result may be related to different variables that converge in this complex infection. These variables include low levels of parasite load and low frequencies of T cells detected during the chronic infection (technical), antigenic variability, tropism and latency of the parasite (parasitological) and genetic background and immune response (host) (60, 69, 82–84).

Tissue injury has been suggested to be mediated by the immune response, which plays a decisive role in the development of chronic ChD (85). For instance, an association between TNF-α-producing cells and cardiac damage has been observed in the heart and plasma samples from patients with chronic ChD with cardiac complications (86, 87), and TNF-α production by T cells from patients with chronic ChD persists and is related to CD8^+^ T cell degranulation (88). Furthermore, patients with advanced-stage ChD disease exhibit a higher proportion of cytotoxic CD8^+^ T cells producing granzyme B and perforin than patients in early stages of the disease (25). Indeed, in chronically infected mice, cytotoxic T cells were observed in cardiac tissue samples, suggesting that these cells are implicated in cardiac damage (31). For instance, studies have suggested that cytotoxic T cells could be related to a mechanism of tissue damage induced by *T. cruzi* infection, because these cells can be detected in tissue from chagasic patients with megaoesophagus or myocardial lesions (28–31). However, it is necessary to elucidate whether, during chronic *T. cruzi* infection, the absence of these molecules —TNF-α, granzyme B, or perforin— could prevent or enhance the Chagasic tissue lesions. Chronically infected mice exhibited a higher percentage of T cells that produce TNF-α than acutely infected mice, and late chronically infected mice showed a higher proportion of cytotoxic CD8^+^ T cells producing granzyme B and perforin than infected mice at 100 dpi, suggesting that our results are similar to previous studies and correlated with the severity of ChD. Recently, despite the chronic infection with *T. cruzi*, a population of CD8^+^ T cells retain functional and cytotoxic activities against the parasite in the tissue (89). Accordingly, in contrast with the classical exhaustion model described for lymphocytic choriomeningitis virus (LCMV) (90), the dysfunctionality of T cells observed in the experimental model of ChD in the present study did not affect all of the functional characteristics of CD8^+^ T cells (i.e., cytotoxic capacity). However, the roles of these populations of T cells in the natural course of *T. cruzi* infection remain to be elucidated.

Classically, inhibitory receptors are associated with processes related to immune regulation, tolerance and the prevention of autoimmunity (23, 56). However, these molecules are also associated with T cell exhaustion during chronic infections (24). Acute experimental models of ChD have shown that the infection induces the transient expression of inhibitory receptors, such as PD-1 (CD279) and CTLA-4 (CD152), on T cells and tissue-infiltrating T cells in the myocardium (91, 92). In addition, acute *T. cruzi*-infected mice treated with blocking antibodies against PD-1 or PD-L1 show reduced parasitemia and parasitism and an increased cardiac inflammatory response and mortality compared to infected mice treated with an isotype control (IgG) (91). In contrast, acute *T. cruzi*-infected mice treated with anti-CTLA-4 show reduced parasitemia and mortality compared to mice treated with an isotype control (IgG) (92). In chronic experimental models of ChD and in patients with a severe form of chronic ChD, a continuous increase in inhibitory receptor expression and co-expression on T cells from peripheral blood and cells from cardiac tissue has been observed (68, 93). Recently, cardiac tissue cells from mice with a chronic infection and heart failure showed high levels of PD-1 and PD-L1 expression at 330 dpi (68). Moreover, mice treated with anti-PD-1 show reduced parasitemia, increased numbers of cardiac T cells with an effector memory phenotype, and increased deterioration of cardiac function compared with infected mice treated with an isotype control (IgG) (68). Remarkably, in the present study, acute *T. cruzi*-infected mice expressed inhibitory receptors, such as 2B4 (CD244), CD160, CTLA-4, and PD-1, at higher levels and exhibited higher co-expression of these molecules at 10 dpi than at 30 dpi, suggesting that the transient increase in inhibitory receptor expression and co-expression on T cells from acute *T. cruzi*-infected mice is related to immune regulation in an acute inflammatory microenvironment. In acutely *T. cruzi*-infected humans, the dynamics of inhibitory receptor expression on T cells are unknown. Given the difficulties of detecting individuals in the acute phase due to non-specific symptoms, inhibitory receptor expression has been evaluated in asymptomatic chagasic patients, and low frequencies of 2B4, CD160, TIM-3, CTLA-4, and PD-1 on CD8^+^ T cells were found compared with those in symptomatic chronic chagasic patients (25). The model described here could reflect the asymptomatic/latent phase, because these molecules are poorly expressed on T cells from *T. cruzi*-infected humans (in the asymptomatic phase) and in mice (at 30 dpi). In addition, we observed a constant increase in the expression of inhibitory receptors and co-expression on CD4^+^ and CD8^+^ T cells from chronically infected mice. Indeed, a higher percentage of T cells expressed CTLA-4 and PD-1 in infected mice at 260 dpi than at 10 dpi. Notably, although these preliminary findings should be further explored, a positive correlation between the percentage of multifunctional T cells and 2B4 and CD160 expression on CD4^+^ and CD8^+^ T cells was observed in acutely infected mice, and a negative correlation between the percentage of multifunctional CD8^+^ T cells and CTLA-4 and PD-1 expression on CD8^+^ T cells was observed in chronically infected mice. Thus, these inhibitory molecules likely play a crucial role in immune regulation (2B4 or CD160) and inhibition (CTLA-4 or PD-1) during acute and chronic *T. cruzi* infection, respectively, and might serve as biomarkers for monitoring the progression of ChD. Intriguingly, although it was postulated that the expression of inhibitory receptors in an acute microenvironment could be related to the regulation of CD4^+^ and CD8^+^ T cell activation, it has been proposed that some of these molecules, such as 2B4 and CD160, could be associated with the optimal activation of T cells (94, 95). However, in acute *T. cruzi* infection, it is necessary to determine whether the expression of these molecules is related to lymphoid regulation or activation. Consequently, blocking the interaction between the inhibitory receptors and their ligands must be carefully analyzed because these molecules play important roles in regulating the hyperactive immune response in both the acute and chronic inflammatory microenvironment.

In conclusion, during acute *T. cruzi* infection with the reticulotropic Y strain, immune activation leads to the generation of antigen-specific multifunctional CD4^+^ Th1 and CD8^+^ Tc1 cells and their regulation by inhibitory receptor co-expression. In contrast, during chronic *T. cruzi* infection, the chronicity of the infection induces a moderate inflammatory infiltrate in colon and liver tissues accompanied with poor T cell effector function that is possibly related to the co-expression of inhibitory receptors on T cells, but this phenomenon does not occur in cytotoxic CD8^+^ T cells. Taken together, these data support our previous study in which we hypothesized that similar to several chronic infectious diseases in humans and murine models, the *T. cruzi* persistence could promotes the dysfunctionality of T cells, and these changes are potentially related to the progression of ChD. Thus, these data constitute a useful model for the identification of immune markers and correlates of protection, and for long-term explorations of new immunotherapy strategies for ChD.

## Ethics Statement

This study was performed in accordance with the ethical standards of the Institutional Animal Care and Use Committee (IACUC, approval FUA-007-14) from the Unidad de Biologá Comparativa (UBA) at Pontificia Universidad Javeriana (PUJ, Bogotá Colombia). All animal studies were conducted in accordance with the Guide for the Care and Use of Laboratory Animals from UBA-PUJ.

## Author Contributions

JM, PL, CP, and AC designed the experiments. JM, PG, PL, and CC performed the experiments. JM, PL, CC, JG, CP, and AC analyzed the data. JM wrote the first draft of the manuscript. All authors contributed to manuscript revision and read and approved the submitted version.

### Conflict of Interest Statement

The authors declare that the research was conducted in the absence of any commercial or financial relationships that could be construed as a potential conflict of interest.
