# Supplementary material for: An Animal Model of Acute and Chronic Chagas Disease With the Reticulotropic Y Strain of Trypanosoma cruzi That Depicts the Multifunctionality and Dysfunctionality of T Cells
Source: Front Immunol. 2019 Apr 26;10:918. doi: 10.3389/fimmu.2019.00918 (PMC6499084; doi:10.3389/fimmu.2019.00918)
Supplement: Supplementary file 1 [file Table_1.docx]

**An animal model of acute and chronic Chagas disease with the reticulotropic Y strain of *Trypanosoma cruzi* that depicts the multifunctionality and dysfunctionality of T cells**

**Jose Mateus^1,2^, Paula Guerrero^1^, Paola Lasso^1,2^, Claudia Cuervo^2^, John Mario González^3^, Concepción J. Puerta^2^, Adriana Cuéllar^1*^**

^1^Grupo Inmunobiología y Biología Celular, Pontificia Universidad Javeriana, Bogotá, Colombia.

^2^Grupo de Enfermedades Infecciosas, Facultad de Ciencias, Pontificia Universidad Javeriana, Bogotá, Colombia.

^3^Grupo de Ciencias Básicas Médicas, Facultad de Medicina, Universidad de los Andes, Bogotá, Colombia.

***Correspondence to:**

Dr. Adriana Cuéllar Ávila, email: [acuellar@javeriana.edu.co](mailto:acuellar@javeriana.edu.co)

Supplementary table

**Supplementary Table.** Detection of *T. cruzi* by cPCR in the colon, heart, liver, skeletal muscle and blood from acutely and chronically infected mice.

| Phase | dpi | Mouse* | Tissue^¥†^ | | | | |
| --- | --- | --- | --- | --- | --- | --- | --- |
|  |  |  | Colon | Heart | Liver | S. muscle | Blood |
| Acute | 10 dpi | R1 | + | + | - | + | + |
|  |  | R2 | + | + | - | - | + |
|  |  | R3 | + | + | - | + | + |
|  |  | R4 | + | + | + | + | + |
|  |  | R5 | + | + | + | + | + |
|  | 30 dpi | R1 | + | + | + | - | + |
|  |  | R2 | + | - | - | - | + |
|  |  | R3 | + | + | - | - | + |
|  |  | R4 | + | + | - | + | + |
|  |  | R5 | - | - | - | + | + |
|  | *Positive results*, n (%) | | 9 (90) | 8 (80) | 3 (30) | 6 (60) | 10 (100) |
| Chronic | 100 dpi | R1 | - | - | - | - | + |
|  |  | R2 | - | - | - | - | - |
|  |  | R3 | - | - | - | - | - |
|  |  | R4 | - | - | - | - | - |
|  |  | R5 | + | - | - | - | + |
|  | 260 dpi | R1 | - | - | + | - | - |
|  |  | R2 | - | + | - | - | - |
|  |  | R3 | - | - | - | + | - |
|  |  | R4 | - | - | - | - | - |
|  |  | R5 | + | + | - | - | - |
|  | *Positive results*, n (%) | | 2 (20) | 2 (20) | 1 (10) | 1 (10) | 2 (20) |

*The column shows the detection of the parasite in each mouse included in the group.

^¥^ For all tissues. Parasite detection was performed by cPCR using gDNA, with the primers S35-S36 and TcH2AF- TcH2AR, as described in the Materials and Methods section.

† Blue color indicates the mouse tissues in which the parasite was detected (+) and red in which it was not detected (-).

dpi, days postinfection
