# Supplementary material for: An Animal Model of Acute and Chronic Chagas Disease With the Reticulotropic Y Strain of Trypanosoma cruzi That Depicts the Multifunctionality and Dysfunctionality of T Cells
Source: Front Immunol. 2019 Apr 26;10:918. doi: 10.3389/fimmu.2019.00918 (PMC6499084; doi:10.3389/fimmu.2019.00918)
Supplement: Supplementary file 2 [file Data_Sheet_1.docx]

**An animal model of acute and chronic Chagas disease with the reticulotropic Y strain of *Trypanosoma cruzi* that depicts the multifunctionality and dysfunctionality of T cells**

**Jose Mateus^1,2^, Paula Guerrero^1^, Paola Lasso^1,2^, Claudia Cuervo^2^, John Mario González^3^, Concepción J. Puerta^2^, Adriana Cuéllar^1*^**

^1^Grupo Inmunobiología y Biología Celular, Pontificia Universidad Javeriana, Bogotá, Colombia.

^2^Grupo de Enfermedades Infecciosas, Facultad de Ciencias, Pontificia Universidad Javeriana, Bogotá, Colombia.

^3^Grupo de Ciencias Básicas Médicas, Facultad de Medicina, Universidad de los Andes, Bogotá, Colombia.

***Correspondence to:**

Dr. Adriana Cuéllar Ávila, email: [acuellar@javeriana.edu.co](mailto:acuellar@javeriana.edu.co)

Supplementary material


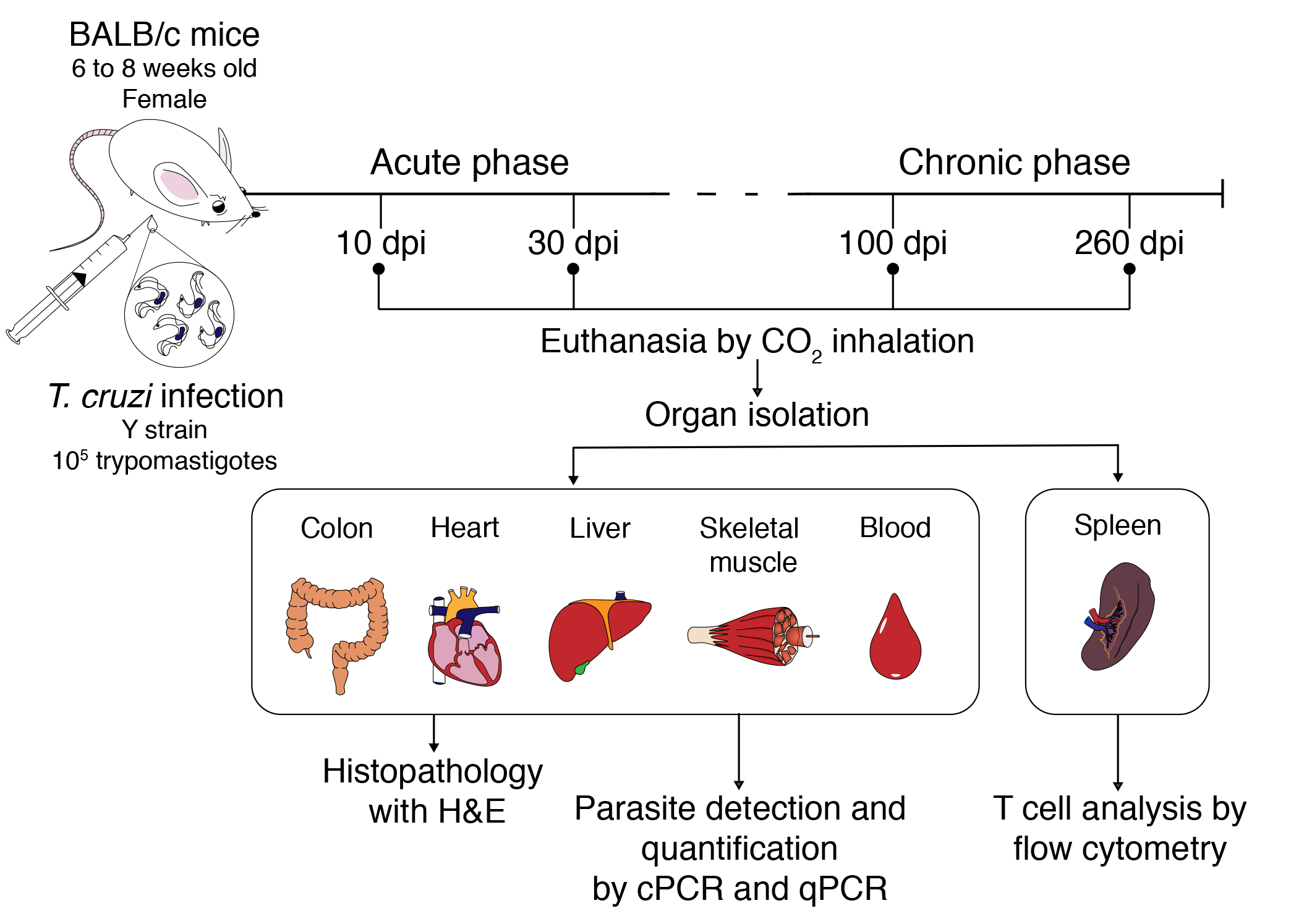


**Supplementary Figure 1. Study design.** The acute and chronic phases were examined in mice that were euthanized at 10 and 30 or 100 and 260 days postinfection (dpi), respectively. Blood and solid tissues, such as colon, heart, liver, and skeletal muscle, were obtained from each mouse for histopathological analysis and parasite detection and quantification. Additionally, the spleen was disaggregated to evaluate the T cell responses in mice with acute and chronic infections.


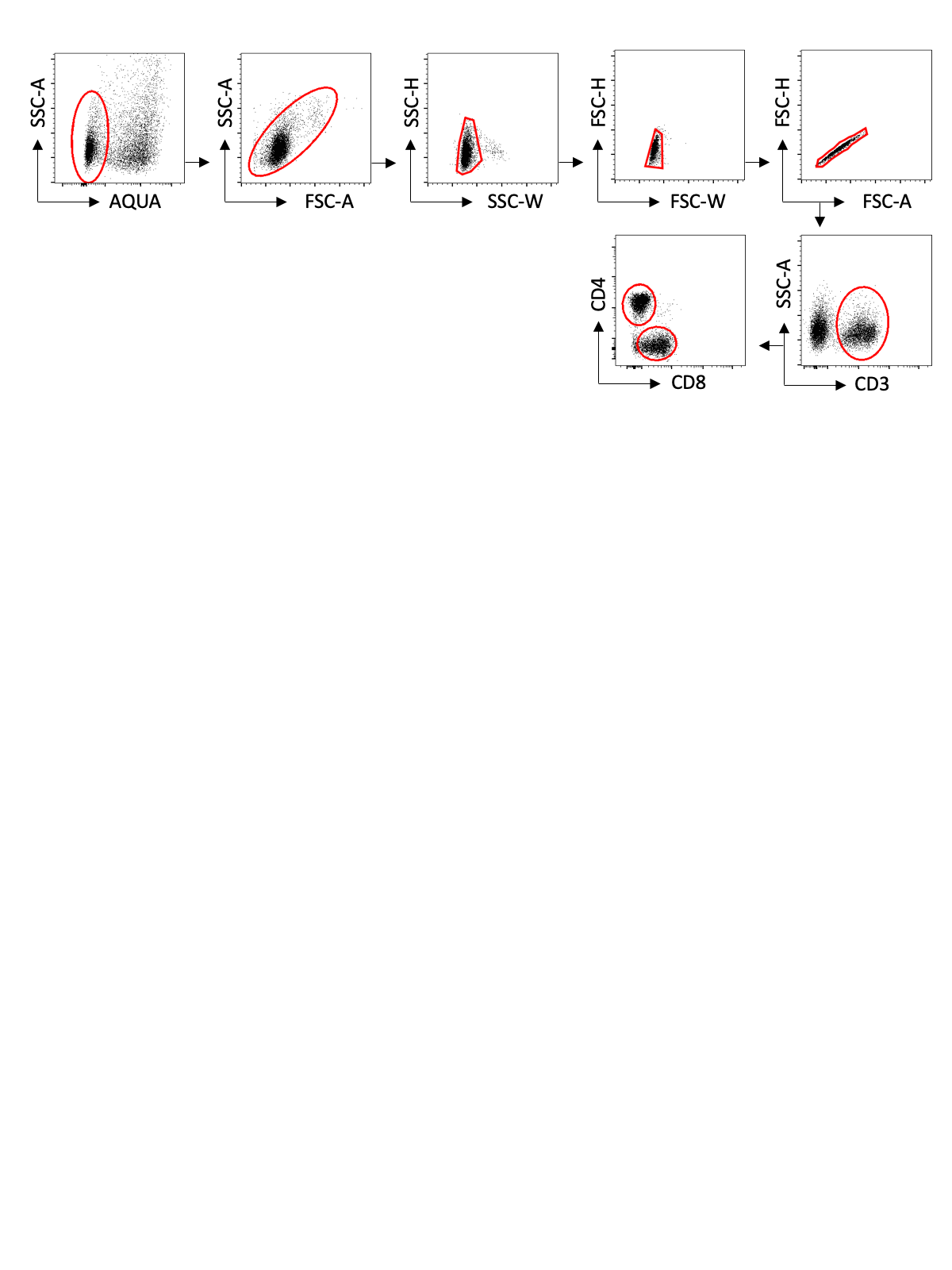


**Supplementary Figure 2. Description of the gating strategy for CD4^+^ and CD8^+^ T cells.** Initially, dead cells were excluded from analysis. Then, lymphocytes were identified in forward scatter (FSC) and side scatter (SSC) plots. The doublets were excluded by the SSC-H vs. SSC-W, FSC-H vs. FSC-W, and FSC-H vs. FSC-A. Next, the CD3^+^ and CD4^+^ or CD8^+^ population was selected.

**Supplementary Figure 3. Dynamic range of the qPCR for *T. cruzi* quantification in mice tissues.** The curve was constructed with different concentrations of *T. cruzi* Y strain gDNA mixed with 50 ng of gDNA from the heart or colon tissue of one uninfected mouse, ranging from 10^-1^ – 10^4^ parasite equivalents per ml. The equation, the coefficient of determination (r^2^), and the efficiency are indicated.

**Supplementary Figure 4. Distribution of the parasitemia and parasite load in tissues from acutely and chronically *T. cruzi***-**infected mice (A)** Parasitemia assessed in tail venous blood from *T. cruzi*-infected mice. **(B)** Parasite loads in the colon, heart, liver, skeletal muscle, and blood samples from mice with acute and chronic *T. cruzi* infections. The parasitemia and the parasite load were evaluated by direct microscopic and qPCR methods, respectively, as described in the Materials and Methods section. The points and vertical lines show the median and range, respectively, of parasites per ml (A) or parasite load (B) in each tissue. ***p* < 0.01, Mann-Whitney U test.

**Supplementary Figure 5. Inflammatory infiltrate and parasite detection/quantification in tissue samples from *T. cruzi*-infected mice**. **(A)** Association between the inflammatory infiltrate and parasite detection in colon, heart, liver, and skeletal muscle by cPCR and/or qPCR in *T. cruzi*-infected mice. Point shows the histopathological scores and parasite detection using one (cPCR or qPCR) or two methods (cPCR and qPCR). The parasite detection by cPCR —using the S35/S36 and the TcH2AF/TcH2AR primers— and qPCR —using the Cruzi 1/Cruzi 2 primers— was performed as described in materials and methods. Fisher's exact test was used to evaluate whether the inflammatory infiltrates were associated with the parasite detection in positive samples by cPCR and qPCR. **(B)** Parasite loads according to inflammatory infiltrate scores in the colon, heart, liver, and skeletal muscle tissues from mice with acute and chronic *T. cruzi* infections. Low inflammatory infiltrates included data from mice with absent or low histopathological scores. The bar graphs show the median parasite equivalent per 50 ng of DNA (LOG_10_) in each tissue from each mouse. The dotted line represents the cut-off for the limit of detectable quantification (LOQ) based on serially diluted *T. cruzi*-spiked tissue DNA as described in the Materials and Methods (0.1 parasite equivalents per 50 ng of DNA). **p* < 0.05, ***p* < 0.01, and ****p* < 0.001, Mann-Whitney U test. OR, odds ratio; CI, confidence interval.

**Supplementary Figure 6. Correlation between the multifunctionality of T cells and the inhibitory receptor expression on T cells.** A correlation analysis was performed between the percentages of Ag-specific CD4^+^ (left panel) and CD8^+^ (right panel) T cells endowed with two and three functions and the percentages of CD4^+^ and CD8^+^ T cells expressing 2B4 or CD160 from mice with acute experimental ChD **(A)** or T cells expressing CTLA-4 or PD-1 from mice with chronic **(B)** experimental ChD. Spearman’s rank correlation test.
